# Prognostic value of computed tomographic findings in acute respiratory distress syndrome and the response to prone positioning

**DOI:** 10.1186/s12890-022-01864-9

**Published:** 2022-02-25

**Authors:** You-Yi Chen, Jerry Shu-Hung Kuo, Sheng-Yuan Ruan, Ying-Chun Chien, Shih-Chi Ku, Chong-Jen Yu, Jung-Yien Chien

**Affiliations:** 1 Division of Pulmonary Medicine, Department of Internal Medicine, National Taiwan University Hospital Yun-Lin Branch, National Taiwan University College of Medicine, No.579, Sec. 2, Yunlin Road., Douliu City, Yunlin County 640 Taiwan (R.O.C.); 2 Thoracic Medicine Center, National Taiwan University Hospital Yun-Lin Branch, National Taiwan University College of Medicine, No.579, Sec. 2, Yunlin Road., Douliu City, Yunlin County 640 Taiwan (R.O.C.); 3 Division of Pulmonary Medicine, Department of Internal Medicine, National Taiwan University Hospital and National Taiwan University College of Medicine, No. 7, Zhongshan S. Rd., Zhongzheng Dist., Taipei City, 100 Taiwan (R.O.C.)

**Keywords:** Prone positioning, Acute respiratory distress syndrome, Computed tomography, Lung imaging, Intensive care unit

## Abstract

**Background:**

Prone positioning enables the redistribution of lung weight, leading to the improvement of gas exchange and respiratory mechanics. We aimed to evaluate whether the initial findings of acute respiratory distress syndrome (ARDS) on computed tomography (CT) are associated with the subsequent response to prone positioning in terms of oxygenation and 60-day mortality.

**Methods:**

We retrospectively included patients who underwent prone positioning for moderate to severe ARDS from October 2014 to November 2020 at a medical center in Taiwan. A semiquantitative CT rating scale was used to quantify the extent of consolidation and ground-glass opacification (GGO) in the sternal, central and vertebral regions at three levels (apex, hilum and base) of the lungs. A prone responder was identified by a 20% increase in the ratio of arterial oxygen pressure (PaO_2_) to the fraction of oxygen (FiO_2_) or a 20 mmHg increase in PaO_2_.

**Results:**

Ninety-six patients were included, of whom 68 (70.8%) were responders. Compared with nonresponders, responders had a significantly greater median dorsal–ventral difference in CT-consolidation scores (10 vs. 7, *p* = 0.046) but not in CT-GGO scores (− 1 vs. − 1, *p* = 0.974). Although dorsal–ventral differences in neither CT-consolidation scores nor CT-GGO scores were associated with 60-day mortality, high total CT-GGO scores (≥ 15) were an independent factor associated with 60-day mortality (odds ratio = 4.07, 95% confidence interval, 1.39–11.89, *p* = 0.010).

**Conclusions:**

In patients with moderate to severe ARDS, a greater difference in the extent of consolidation along the dependent-independent axis on CT scan is associated with subsequent prone positioning oxygenation response, but not clinical outcome regarding survival. High total CT-GGO scores were independently associated with 60-day mortality.

**Supplementary Information:**

The online version contains supplementary material available at 10.1186/s12890-022-01864-9.

## Background

Prone positioning has been strongly recommended for patients with acute respiratory distress syndrome (ARDS) [1] due to its physiological and survival benefits demonstrated in clinical trials [2, 3]. Prone positioning enables redistribution of the lung weight along the dependent-nondependent axis to improve dorsal lung aeration, ventilation-perfusion relations, and lung mechanics. Oxygenation improvement and mortality reduction are the main purposes to implement prone positioning in patients with ARDS [3–6]. Prone positioning combined with lung protective ventilation leads to less overdistension in nondependent lung regions and less cyclical opening and closing-related lung injury in dependent regions, thereby explaining the mechanism that reduces mortality [7]. Despite these benefits, the literature has demonstrated several adverse events, such as incidental extubation, removal of intravascular catheters, airway obstruction, facial edema, pressure sores and increased sedation use [8, 9]. Although the only absolute contraindication for performing the procedure is unstable fracture of the spine, prospective observational studies found a low application rate of prone positioning even in severe ARDS patients [10]. Findings from the "Lung Safe" ARDS epidemiology study revealed that only 60.2% of all patients with ARDS were clinician-recognized. Because of delayed diagnosis, the rate of adherence to lung-protective protocols was as low as 35.1%, and the use of adjunctive treatments was relatively low but increased with ARDS severity as the recognition of ARDS improved [11]. Therefore, the overall use rate of prone positioning was low across all ARDS patients (the rate ranged from 1% for mild ARDS to 16.3% for severe ARDS).

In clinical practice, being able to predict the response of prone positioning may aid in decision-making when selecting a management strategy for ARDS. Several studies have attempted to predict the response of prone positioning using different modalities. Lung ultrasound was able to monitor regional aeration changes but failed to demonstrate good correlation with oxygenation improvement from prone positioning in multicenter studies [12, 13]. Computed tomography (CT) has the advantage of being able to characterize the spatial distribution of consolidation and ground-glass opacities (GGOs) in the lungs. In ARDS, CT typically shows diffuse opacification with dense consolidation in the dependent regions, widespread ground-glass attenuation, and hyperexpanded lung parenchyma in the nondependent part of the lung. Previous studies have suggested that more opacification in the dependent lung regions when in the supine position is associated with more recruitable lung volume when changed to the prone position [14–16]. However, data regarding the prognostic value of CT in ARDS are scarce.

Several CT scoring models have been proposed in ARDS in different clinical settings. Ichikado et al. used a CT scoring system to predict the prognosis of ARDS by calculating the extent of the fibroproliferative changes using thin-section CT to predict prognosis in patients with a clinically early stage of ARDS and drug-associated ARDS [17–19]. The scoring system graded CT scans in terms of the extent of normal attenuation, ground-glass attenuation, consolidation, coexistence of traction bronchiolectasis or honeycombing. A higher CT score represented extensive fibroproliferative changes and was associated with poor prognosis. In a recent COVID-19 study, the Ichikado CT score was found to be an independent predictor of both the requirement for invasive mechanical ventilation and all-cause mortality in patients hospitalized with COVID-19 pneumonia. However, little is known about whether radiographic morphology patterns on chest CT are predictive of the response to prone positioning and survival in patients with ARDS [20, 21].

We hypothesized that certain radiographic patterns of disease extent in chest CT correlate with the amount of recruitable lung volume when in the prone position. This might be useful to predict the response of prone positioning and survival. Therefore, we aimed to explore the association between semiquantified CT lung morphology and the response to prone positioning regarding gas exchange and survival in patients with moderate to severe ARDS.

## Methods

### Study design, setting, and participants

The Institutional Review Board of the National Taiwan University Hospital approved this study (201312099RINB). The study complied with the Declaration of Helsinki. All consecutive mechanically ventilated patients admitted to the medical intensive care unit (ICU) at the National Taiwan University Hospital during the period from October 2014 to November 2020 were screened. We retrospectively recruited patients with moderate to severe ARDS who underwent prone positioning therapy during the study period. Patients with whole-lung high spatial resolution CT (HRCT) scans within 72 h and prior to the first prone-positioning session were included for analysis, while patients who did not have HRCT scans or the time interval between HRCT scans and first prone positioning exceeding 72 h were excluded. The diagnosis of ARDS was established according to the following Berlin definition: (1) timing: within 1 week of a known clinical insult or new or worsening respiratory symptoms; (2) chest imaging: bilateral opacities, not fully explained by effusions, lobar/lung collapse, or nodules; (3) origin of edema: respiratory failure not fully explained by cardiac failure, fluid overload or hydrostatic edema, which was excluded using echocardiography if no risk factor was present; (4) oxygenation, classified into 3 categories according to P/F (arterial oxygen pressure/fraction of inspired oxygen) ratio: mild ARDS with a P/F ratio between 200 and 300, moderate ARDS with a P/F ratio between 100 and 200, severe ARDS with a P/F ratio less than 100 and a positive end-expiratory pressure ≥ 5 cm H_2_O. All the patients received moderate to deep sedation and/or paralysis as needed for the achievement of ventilatory synchrony since moderate or severe ARDS was impressed.

### Data collection

Clinical information, laboratory results and lung mechanics, including sex, age, comorbidities (including stroke, cardiovascular disease, chronic kidney disease, diabetes mellitus, cancer, autoimmune disease, chronic liver disease, chronic lung disease), body mass index, serum albumin, hemoglobin, creatinine levels, Sequential Organ Failure Assessment (SOFA) score, Simplified Acute Physiology Score (SAPS) II, cause of acute respiratory failure, duration of mechanical ventilation and dynamic parameters of lung mechanics, were collected.

### Prone positioning and response

Prone positioning was performed according to a standardized protocol for at least 12 h [3, 22]. The median duration of prone positioning in this study was 16 h (range 12–24). The median number of sessions administered to the patients was 2 (range 1–22). Arterial blood gases were analyzed prior to turning patients to the prone position and 1–5 h (median: 2 h) after turning them to prone position. Patients with a P/F ratio increasing by at least 20% or ≥ 20 mmHg after prone positioning were classified as “responders”, while those not meeting these criteria were classified as “nonresponders”. The responder definition threshold referred to previous studies. [23–27]

### Measurements of CT scores

Whole-lung high spatial resolution CT images obtained within 72 h and prior to the first prone-positioning session were analyzed. Sections throughout the chest were displayed at 5 mm intervals with the patient in the supine position. To quantify the extent of pulmonary abnormalities, the results of the CT scans were evaluated using a modified method previously described by Goodman et al.[21] In brief, three representative levels of CT were selected for analysis: the apex (top of the upper aortic arch), the hilum (first section below the carina), and the base (2 cm above the highest hemidiaphragm). Each transverse image was further divided into three sections: an anterior third (sternal), middle third (central), and posterior third (vertebral). The left and right lung were analyzed individually. This equated to 9 anatomical locations for each lung, so together, the lungs consisted of a total of 18 areas. Each of the 18 areas was rated according to the extent that a normal lung (NL), consolidation (CO) and ground-glass opacification (GGO) were present, using a six-point scale of 0–5 [28]. Higher CT scores indicated a greater extent of lung involvement (0: the morphologic features were essentially absent, 1: 1–20% area involved, 2: 21–40% area involved, 3 = 41–60% area involved, 4: 61–80% area involved, 5: 81–100% area involved). The sum for each area had to equal five. For each pattern, the CT scores of both lungs per section and per level were summed. Differences between the CT scores of the vertebral and sternal sections were used as an index of dorsal–ventral lung involvement (dorsal–ventral difference). No additional correction or weight was applied to calculate the morphologic score between different levels. The HRCT scans were evaluated by two chest physicians who were blinded to the clinical outcomes. A representative case is demonstrated in Additional file 1: Fig. S1. The primary endpoint was oxygenation after prone positioning. We also evaluated survival outcomes, and patients surviving on the 60^th^ day after prone positioning were defined as survivors. Twenty-eight-day survival and ICU survival were also analyzed as sensitivity analyses.

### Statistical analysis

Categorical variables were compared using the chi-squared test or Fisher’s exact test, where appropriate, and differences in continuous variables were analyzed using the independent t test or Mann–Whitney rank-sum test, depending on the data distribution. Data are presented as either the number (percentage) of patients, the mean ± standard deviation or the median (with interquartile range, IQR). Survival analysis was performed using the Kaplan–Meier method, and significance was assessed using the log-rank test. Multivariate logistic regression analysis with the forced-in method was used to determine the independent variables that were predictive of prone response and 60-day mortality. A *p* value of < 0.05 was considered a statistically significant difference; all tests were two-tailed.

## Results

### Participants and characteristics

**Fig. 1:**
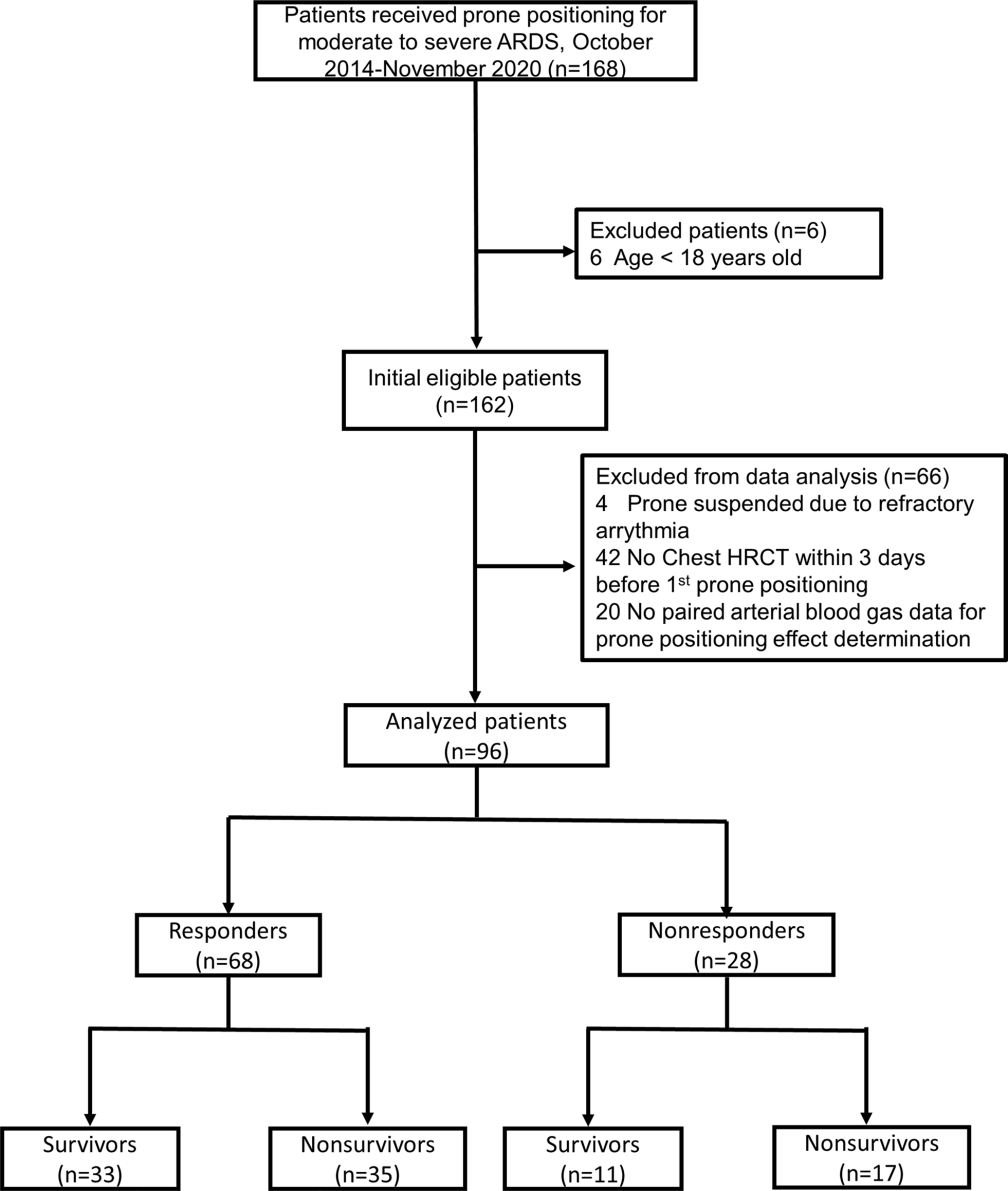
Flow chart of this observational study

During the study period, 168 patients received prone positioning for moderate to severe ARDS in our ICUs, of whom 162 patients were included. A total of 96 patients who fulfilled the study criteria were finally analyzed; among them, 68 (70.8%) patients were responders, and 28 (29.2%) were nonresponders (Fig. 1). The responders showed a significant increase in the P/F ratio (from 95.1 ± 34.8 to 184.4 ± 67.5 mmHg, *p* < 0.001) after prone positioning, while the P/F ratio among the nonresponders changed marginally (from 103.5 ± 31.4 to 102.2 ± 24.2 mmHg, *p* = 0.798). Table 1 shows that the demographic data, etiology of ARDS, disease severity scores and initial P/F ratios did not differ significantly between the responders and nonresponders.

**Table 1:** Clinical characteristics of the patients with acute respiratory distress syndrome, categorized according to prone positioning response and survival

Characteristics	Response to prone positioning			Survival		
	Responders (n = 68)	Nonresponders (n = 28)	*p* value^†^	Survivors (n = 44)	Nonsurvivors (n = 52)	*p* value^‡^
Age, years	66.9 ± 15.8	64.4 ± 16.9	0.501	65.7 ± 16.7	66.5 ± 15.6	0.803
Male sex	41 (60.3)	15 (53.6)	0.544	30 (68.2)	26 (0.5)	0.072
Body mass index	23.7 ± 4.2	25.2 ± 6.7	0.189	25.2 ± 5.9	23.2 ± 4.15	0.054
*Etiology of ARDS*						
Pneumonia	30 (44.1)	13 (46.4)	0.836	20 (45.5)	23 (44.2)	0.904
Aspiration	14 (20.6)	4 (14.3)	0.472	9 (20.5)	9 (17.3)	0.694
Sepsis	13 (19.2)	5 (17.9)	0.886	8 (18.2)	10 (19.2)	0.896
Other	11 (16.2)	6 (21.4)	0.540	7 (15.9)	10 (19.2)	0.671
*Comorbidity*						
Remote stroke	13 (19.1)	2 (7.1)	0.142	10 (22.7)	5 (9.6)	0.080
Cardiovascular disease	17 (25)	4 (14.3)	0.248	11 (25)	10 (19.2)	0.496
Chronic kidney disease	1 (1.5)	9 (32.1)	0.159	6 (13.6)	4 (7.7)	0.342
Diabetes mellitus	36 (52.9)	12 (42.9)	0.369	25 (56.8)	23 (44.2)	0.219
Cancer	25 (36.8)	8 (28.6)	0.442	11 (25)	22 (42.3)	0.075
Immunocompromised	11 (16.2)	5 (17.9)	0.841	5 (11.4)	11 (21.2)	0.200
Hepatic disease	4 (5.9)	2 (7.1)	0.817	3 (6.8)	3 (5.8)	0.832
Pulmonary disease	8 (11.2)	7 (25)	0.105	9 (20.5)	6 (11.5)	0.231
*Respiratory mechanics*						
Tidal volume (ml)	448.8 ± 155.4	437.6 ± 108.2	0.731	421.9 ± 103.6	465.5 ± 167.4	0.137
Tidal volume (ml/kg of PBW)	7.9 ± 2.7	8.2 ± 7.3	0.550	7.4 ± 1.9	8.4 ± 2.9	0.160
Respiratory frequency (breathes/min)	23.0 ± 4.7	22.5 ± 6.0	0.655	21.9 ± 4.8	23.6 ± 5.3	0.110
Dynamic driving pressure (cmH_2_O)	15.3 ± 2.7	15.7 ± 4.1	0.594	15.1 ± 3.2	15.6 ± 3.2	0.449
PEEP (cmH_2_O)	11.8 ± 2.5	12.0 ± 2.0	0.745	12.2 ± 2.5	11.6 ± 2.3	0.186
Plateau pressure (cmH_2_O)	27.2 ± 3.5	27.6 ± 3.6	0.485	27.5 ± 4.2	27.4 ± 2.9	0.836
Mean airway pressure (cmH_2_O)	17.7 ± 2.9	17.9 ± 2.5	0.422	17.8 ± 2.9	17.8 ± 2.7	0.136
Compliance (mL/cmH_2_O)	30.6 ± 12.6	30.6 ± 12.7	0.998	29.6 ± 11.6	31.5 ± 13.5	0.452
Mechanical power (J/min)	26.4 ± 8.3	25.5 ± 6.1	0.623	24.2 ± 6.8	27.8 ± 8.1	0.021
SAPS II score	52.5 ± 16.9	50.9 ± 15.8	0.667	47.6 ± 12.9	55.8 ± 18.3	0.016
SOFA score	9.5 ± 3.7	9.8 ± 3.5	0.733	9.1 ± 3.3	9.9 ± 3.8	0.277
Baseline PaO_2_/FiO_2_ ratio	86.4 ± 26.2	95.4 ± 26.2	0.128	92.4 ± 30.3	86.2 ± 22.5	0.254
Vasopressor	56 (82.4)	23 (82.1)	0.980	33 (75)	46 (88.5)	0.085
Total CT-consolidation scores*	24.5 (14–31)	21.5 (8–30.5)	0.278	24.5 (12–33)	23 (12.5–30.5)	0.699
Total CT-GGO scores*	12.5 (2.5–37.5)	23.5 (5–46)	0.376	9 (1–26)	25 (11–46)	0.001

Data are presented as the no. (or %) or mean ± standard deviation

*Data are presented as the median with the IQR

^†^Comparisons between responders and nonresponders

^‡^Comparisons between survivors and nonsurvivors

Abbreviations: FiO2, fraction of oxygen in the inspired gas; PaO2, arterial oxygen pressure*;* SAPS, Simplified Acute Physiology Score; SOFA, Sequential Organ Failure Assessment

With respect to 60-day overall survival, 44 (45.8%) patients were survivors, and 52 (54.2%) were nonsurvivors. Among the survivors, after prone positioning, the mean P/F ratio increased significantly from 102.2 ± 40.7 to 175.4 ± 70.2 mmHg (*p* < 0.001); likewise, among the 52 nonsurvivors, the mean P/F ratio increased significantly from 93.6 ± 26.7 to 147.7 ± 66.4 mmHg (*p* < 0.001). There were no differences in the etiology of ARDS, comorbidities, baseline P/F ratio or vasopressor use between the survivors and nonsurvivors; nevertheless, nonsurvivors presented with higher severity of illness in terms of the SAPS II score but not the SOFA score (Table 1).

### Semiquantified computer tomographic parenchymal abnormalities

**Fig. 2:**
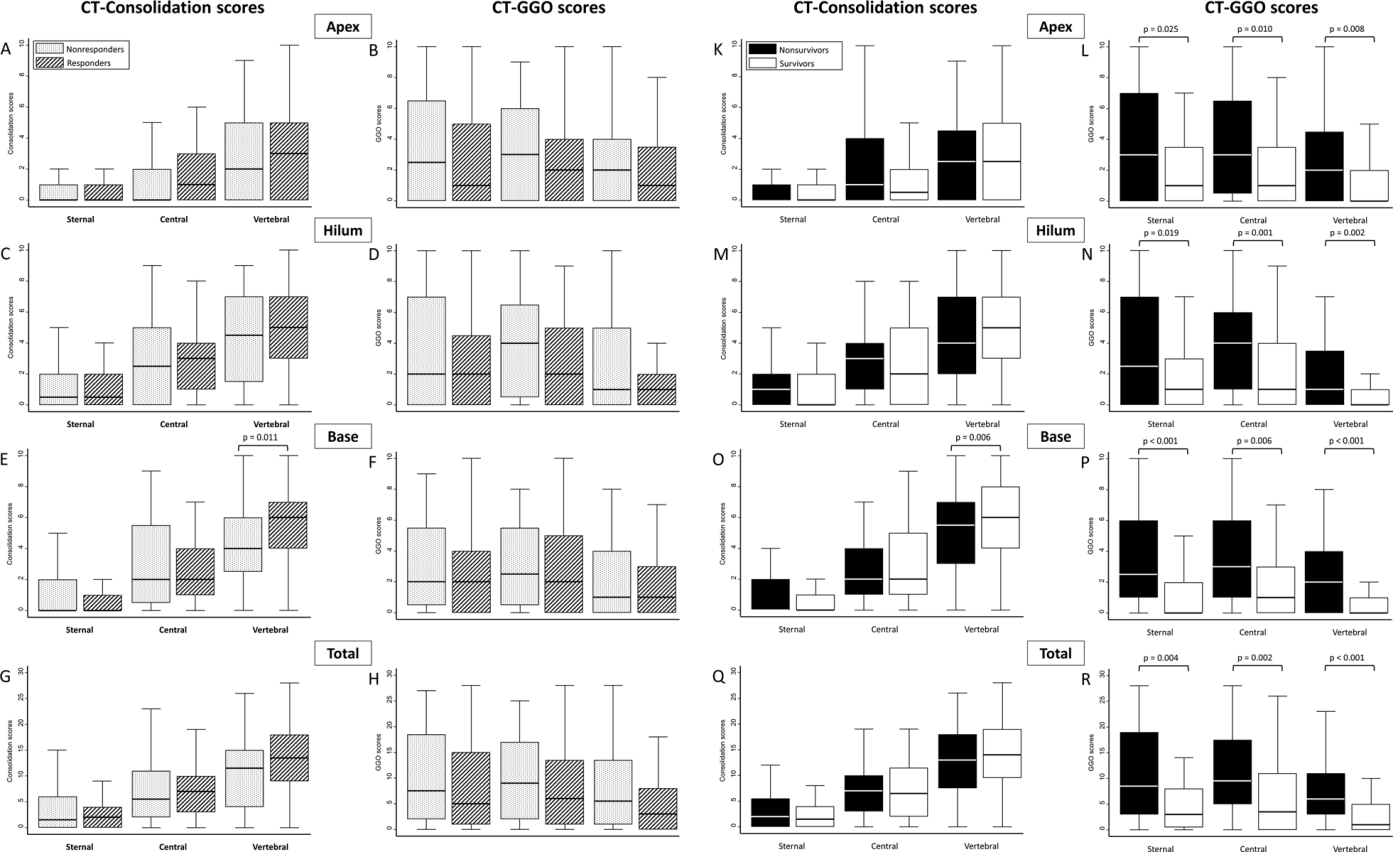
Distribution of computed tomographic (CT) consolidation scores and CT ground-glass opacification (GGO) scores in the apex level (Panel **A**, **B** and Panel **K**, **L**), hilum level (Panel **C**, **D** and Panel **M**, **N**), base level (Panel **E**, **F** and Panel **O**, **P**) and total lung (Panel **G**, **H** and Panel **Q**, **R**), further classified according to the different lung sections, in responders versus nonresponders and survivors versus nonsurvivors

**Fig. 3:**
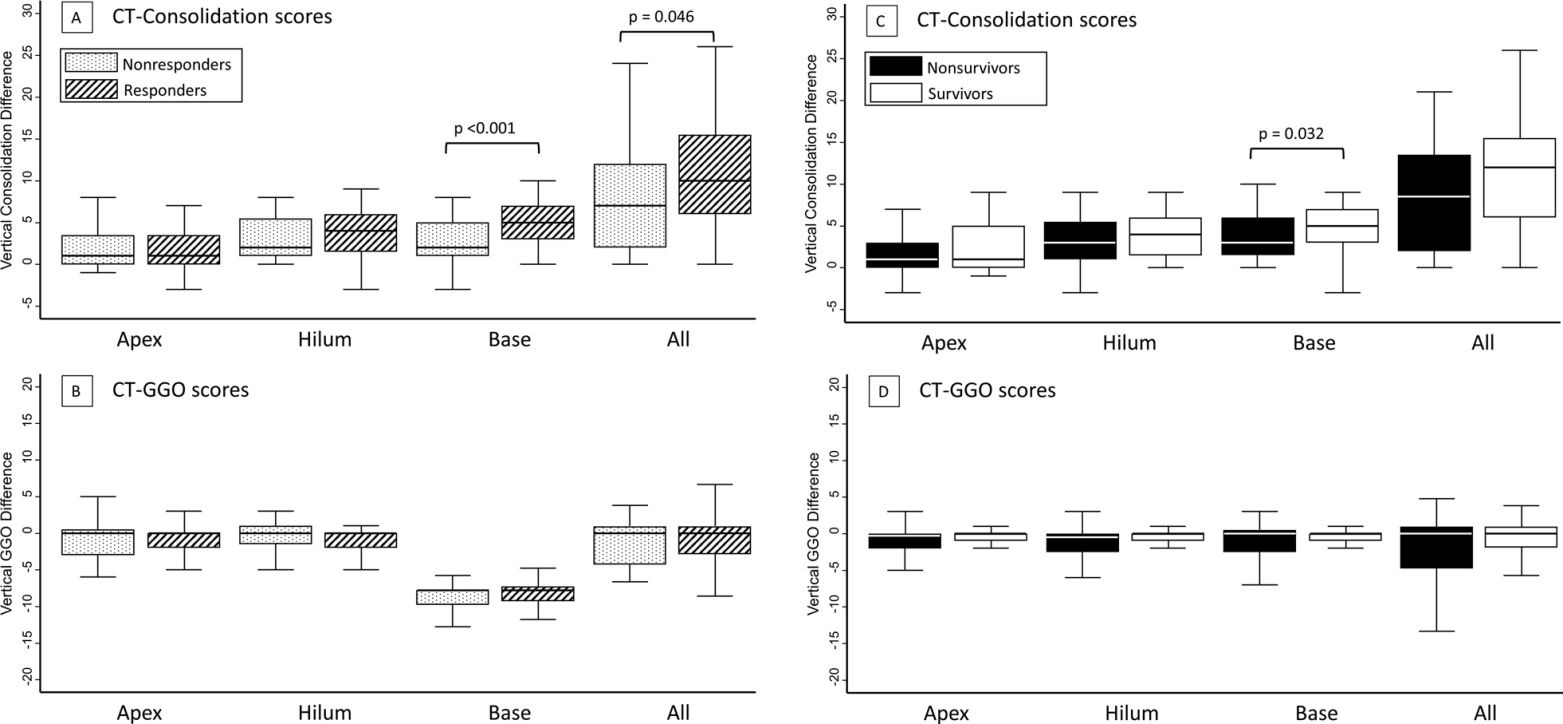
Dorsal–ventral difference in computed tomographic (CT) consolidation and ground-glass opacification (GGO) scores among responders and nonresponders (Panel **A**, **B**), survivals and nonsurvivors (Panel **C**, **D**)

CT-consolidation scores were higher in the vertebral section among responders than among nonresponders, particularly at the lung base level (6, 4–7 vs. 4, 2.5–6, *p* = 0.011), but the distribution of GGOs was similar between responders and nonresponders (Fig. 2). Regarding differences in dependent-nondependent axis distribution, there were significantly greater dorsal–ventral differences in CT-consolidation scores in responders than in nonresponders (10, 6–15.5 vs. 7, 2–12; *p* = 0.046), particularly at the lung base level (5, 3–7 vs. 2, 1–5; *p* < 0.001) (Fig. 3). Multivariate logistic regression analysis showed that dorsal–ventral differences in CT-consolidation scores at the lung base level were an independent predictor of responders (adjusted odds ratio, aOR = 1.35, 95% CI 1.12–1.63, *p* = 0.002, Additional file 1: Table S1).

Compared to nonsurvivors, CT-consolidation scores were also higher in the vertebral section among survivors at the lung base level (6, 4–8 vs. 5.5, 3–7, *p* = 0.006). Furthermore, nonsurvivors had significantly higher CT-GGO scores than survivors in all three sections: sternal (8.5, 3–19 vs. 3, 0.5–8; *p* = 0.004), central (9.5, 5–17.5 vs. 3.5, 0–11; *p* = 0.002), and vertebral (6, 3–11 vs. 1, 0–5; *p* < 0.001) lung sections, as well as the summation (25, 11–46 vs. 9, 1–26; *p* = 0.001) (Table 1 and Fig. 2). Survivors had greater dorsal–ventral differences in CT-consolidation scores at the lung base level than nonsurvivors (5.5, 3–7 vs. 2.5, 1.5–4.5; *p* = 0.036), but this was not true for the apex and hilum levels. In terms of dorsal–ventral differences in CT-GGO scores, no significant differences were found between survivors and nonsurvivors.

The power of using total CT-GGO scores to predict mortality was assessed with receiver operator characteristic (ROC) curve analysis, and the area under the ROC curve (AUC) was 0.69 (95% CI 0.59–0.80, Additional file 1: Figure S2). Using a cutoff value of CT-GGO scores ≥ 15, the sensitivity, specificity, positive-predictive and negative-predictive value for survivors was 69.2%, 68.2%, 72% and 65.2%, respectively. Multivariate logistic regression analysis indicated that the dorsal–ventral consolidation difference was not associated with survival, but CT-GGO scores ≥ 15 were independently associated with 60-day mortality (aOR: 4.07, 95% CI 1.39–11.89; *p* = 0.010) after adjusting for patient characteristics, comorbidities, disease severity, mechanical power and dorsal–ventral differences in CT-consolidation scores at the lung base (Table 2).

**Table 2:** Logistic regression to evaluate the association between 60-day overall survival and a CT ground-glass opacification score of ≥ 15 points

Model	CT ground-glass opacification score ≥ 15 points	
	Odds ratio (95% CI)	*p* value
Model 1: unadjusted	4.82 (2.03–11.46)	< 0.001
Model 2: adjusted for age, sex and BMI	4.39 (1.77–10.89)	0.001
Model 3: further adjusted for cancer and remote stroke	5.68 (2.08–15.48)	0.001
Model 4: further adjusted for the SAPS II score	5.12 (1.85–14.17)	0.002
Model 5: further adjusted for mechanical power and dorsal–ventral differences in the CT-consolidation scores at the lung base	4.07 (1.39–11.89)	0.010

Abbreviations: CT, computed tomography; SOFA, Sequential Organ Failure Assessment; SAPS, Simplified Acute Physiology Score

### Outcome analysis

**Fig. 4:**
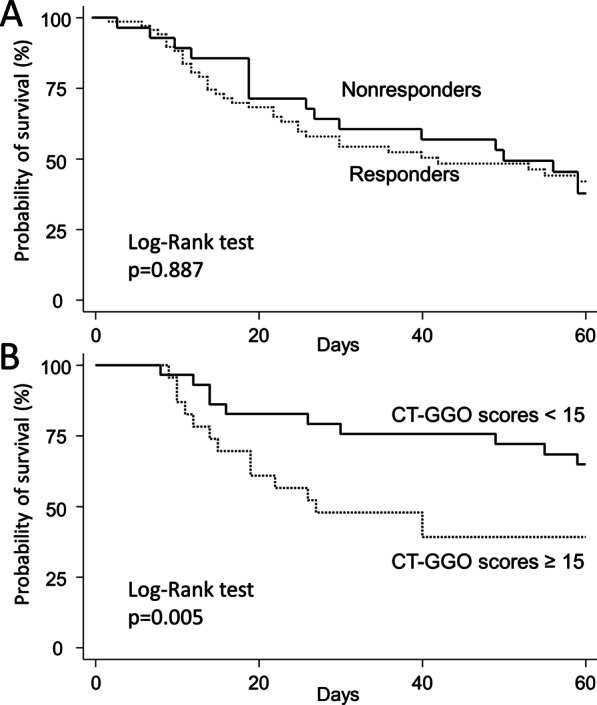
Kaplan–Meier plots with the log-rank test for probability of 60-day survival, according to response of prone positioning (Panel **A**), and total computed tomographic ground-glass-opacification (CT-GGO) scores (Panel **B**)

Table 3 shows no difference in important clinical outcomes between the responders and nonresponders, including the duration of mechanical ventilation, length of ICU stay, proportion of liberation from mechanical ventilator, ICU mortality and 60-day mortality. Furthermore, survival analysis showed that 60-day mortality was similar between responders and nonresponders (51.5% vs. 60.7%, *p* = 0.887 by log-rank test, Fig. 4A). However, compared to patients with lower CT-GGO scores, those with higher CT-GGO scores (≥ 15 points) had significantly higher 28-day and 60-day mortality (34.8% vs. 72%; *p* = 0.005, Fig. 4B) and ICU mortality (Additional file 1: Figure S3).

**Table 3:** Outcomes of patients with acute respiratory distress syndrome following prone positioning

Outcomes	Response to prone positioning		
	Responders (n = 68)	Nonresponders (n = 28)	*p* value†
Length of MV, days	19 (12–38)	19 (12–38)	0.079
MV liberation rate (%)	27 (39.7)	9 (32.1)	0.487
Length of ICU stay, days	22 (12.5–40)	29 (19–49.5)	0.137
ICU mortality (%)	36 (52.9)	19 (67.9)	0.179
60-Day overall mortality (%)	35 (51.5)	17 (60.7)	0.409

Data are presented as the no. (or %) or median (with the IQR)

^†^Comparisons between responders and nonresponders

Abbreviations: CT, computed tomography; MV, mechanical ventilation; ICU, intensive care unit

## Discussion

In the present study, the association between the radiographic disease extent, subsequent prone positioning response and clinical outcome were investigated. We found a greater difference in the extent of consolidation along the dependent-independent axis on CT scan was associated with subsequent prone positioning oxygenation response but not survival. High total CT-GGO scores were independently associated with 60-day mortality. In complex clinical cases, where weighing the pros and cons of prone positioning is needed, a large difference in the extent of consolidation along the dependent-independent axis at the lung base may give rise to a higher likelihood of a significant response, whereas patients who presented with diffuse GGO on HRCT had a lower probability of gaining a survival benefit from prone positioning.

Based on previous roentgenographic studies, ARDS typically has diffuse but heterogeneous regional changes, particularly in the early stages of the disease.[29] The most prevalent radiographic pattern in ARDS is air-space consolidation and ground-glass attenuation, which are not homogeneously distributed but exhibit gravitation-dependent differences, with more consolidation in the dependent regions resulting from compressive gravitational forces.[30] Prone positioning shifts the dorsal lung regions ventrally and could improve gaseous exchange, lead to a more homogeneous distribution of transpulmonary forces through the lung parenchyma, and improve survival [31, 32]. Our results support the hypothesis that a greater difference in the extent of consolidation along the dependent-independent axis could be a helpful tool to predict prone responders in patients with moderate to severe ARDS.

In acute respiratory failure, CT scans can precisely quantify the amounts of nonaerated lung regions and has the advantage of being able to characterize the spatial distribution of consolidation and GGOs in the lungs[33]. However, one prospective study by Papazian et al.[34] failed to demonstrate any distinctive morphological lung features or pattern, measured with CT scanning, capable of predicting the response of prone positioning in mixed ICU patients. We conducted this study in a medical ICU rather than a mixed medical and surgical ICU, and each prone-positioning session was performed for at least 12 h or more to optimize the effect of prone positioning according to several well-designed RCTs published in recent years [3, 35, 36]. In addition, a responder in this study was defined by an increase in the P/F ratio of at least 20% or PaO_2_ by ≥ 20 mmHg according to correlative previous studies [23, 26, 27] rather than the 33% increase used in Papazian’s study. In this study, we demonstrated an easy-to-use scoring system by CT with good performance to predict response and survival following prone positioning.

Despite the difference in the extent of consolidation along the dependent-independent axis predicted subsequent response to prone positioning, it was uncorrelated with survival. Whether improved gas exchange during prone positioning was associated with better survival outcomes remained uncertain. By analyzing prospectively collected data from the PROSEVA trial, researchers found that prone positioning-induced oxygenation improvement did not predict improved survival [3, 26]. Similar to previous studies [37], our study also found that oxygenation improvement from prone positioning was unrelated to survival. In contrast, the improvement in carbon dioxide elimination was reported to be associated with clinical outcome rather than oxygenation improvement after prone positioning [24] but was not evaluated in the present study.

GGOs on CT might reflect an increase in interstitial fluid or protein accumulation. A high extent of GGO implies diffuse alveolar involvement and correlates with higher mortality when combined with the presence of fibrotic changes [17, 18, 38]. In contrast to previous studies, our investigation revealed that the presence of extensive GGOs on CT (≥ 15 points), regardless of fibrotic change, led to a 4.07-fold increase in the odds of 60-day mortality. In exploring the association between high GGO extent and worse 60-day survival probability, we found that patients with a high GGO extent gained little lung mechanical improvement following prone positioning (Additional file 1: Tables S3 and S4). Compared to focal ARDS, diffuse ARDS may show a distinct response to PEEP, recruitment maneuvers and prone positioning [39, 40]. In moderate to severe COVID-19, the GGO extent was found to correlate with higher levels of certain inflammatory cytokines and cytokine storms.[41] Therefore, we assumed that a high intensity of GGOs may imply that the patient presented with a hyperinflammatory phenotype of ARDS, which was associated with a higher severity of lung injury, more impaired respiratory mechanics and a worse chance of survival [42, 43]. Comprehensive inflammatory cytokine profiling combined with radiographic morphology could be a potential surrogate marker of the hyperinflammatory phenotype, inferring better prone response prediction and a more precise treatment plan.

Several imaging techniques have been investigated in ARDS, including the chest radiograph severity score, lung ultrasound and electrical impedance tomography (EIT). The radiographic assessment of lung edema (RALE) score estimates the severity of ARDS by calculating the extent of pulmonary edema on chest radiograph, which has been found to be independently associated with poor oxygenation and worse survival [44]. However, application of the RALE score may be challenging in patients who have extensive atelectasis, pleural effusion or cardiomegaly. Furthermore, the score is unable to estimate the vertical gradient of lung collapse and/or flooding of dorsal airspaces. Lung ultrasound can monitor regional aeration changes and the existence of hyperinflation but fails to demonstrate good correlation with the oxygenation response of prone positioning [13, 45–47]. EIT detects the pulmonary ventilation distribution continuously and is useful in monitoring lung compliance [48–50]. However, EIT only provides a cross-sectional lung-region evaluation, which may vary from that for whole lungs. Although CT involves more radiation exposure and a greater patient-transportation risk than standard chest films, CT has better imaging quality for characterization and quantification of the extent of lung disease. Distinct imaging modalities should be complementary, and integrating different techniques can allow a more comprehensive understanding of the pathogenesis and pathophysiology of ARDS, ensuring optimal personized treatment.

This study has several limitations. First, owing to the retrospective nature of the study, data from some patients might be missing, and the study was susceptible to selection bias because of the specific time criteria for CT scans. Second, this study only aimed to investigate the association between the CT scores and the response after prone positioning; thus, the validation of the predictive value of the CT scores needs further large-scale prospective studies. Third, this was a single-institution study consisting of a relatively small number of patients from the medical ICU; nevertheless, the cohort was therefore less affected by the pulmonary complications seen with surgery or trauma. Fourth, the cumulative fluid balance was not recorded; therefore, the impact of fluid balance on the extent of opacities or the prone response was inaccessible. Finally, there was a time gap between CT scans and first prone positioning, as we analyzed retrospectively, and the disease course and the difference in ventilator parameters may interfere with the morphology-function correlation. We explored the ventilator setting was similar in terms of tidal volume, respiratory frequency, dynamic driving pressure, PEEP, mean airway pressure and mechanical power. There was significantly worse arterial blood gas immediately before the first prone positioning regarding the PaO2/FiO2 ratio, which explained the clinical deterioration in condition that required further prone positioning therapy (Additional file 1: Table S5).

Precision and personalized medicine in ARDS is fundamentally challenging because of diverse etiologies and complex pathophysiologies. Although strict ventilation strategies and prone positioning are proposed to be applied universally in moderate to severe cases, the adherence rate to recommended treatment remains consistently low. This study provides evidence that an easy-to-use scoring system for CT scans within 72 h before prone positioning is an independent predictor of responder and all-cause in-hospital mortality in patients with moderate to severe ARDS despite the etiology. Utilizing the CT score may aid clinical physicians in anticipating the adjuvant prone positioning response and in distinguishing patients with a potentially poor prognosis who might need additional intervention. Further large prospective studies focusing not only on gas exchange but also on lung mechanics and regional perfusion/ventilation changes during prone positioning are needed to validate our results.

## Conclusions

In patients with moderate to severe ARDS, a greater difference in the extent of consolidation along the dependent-independent axis on CT scan is associated with subsequent prone positioning oxygenation response, but not clinical outcome regarding survival. High total CT-GGO scores are independently associated with 60-day mortality.

## Supplementary Information

**Additional file 1.** **Table S1.** Logistic regression to evaluate the association between prone-responders and dorsal-ventral differences in CT-consolidation scores at the lung base. **Table S2.** Ventilator setting and results of arterial blood gas measurement before and after the 1st prone position between responders and nonresponders. **Table S3.** Ventilator setting and results of arterial blood gas measurement before and after the 1st prone position between survivors and nonsurvivors. **Table S4.** Ventilator setting and results of arterial blood gas measurement before and after the 1st prone position between the high and low GGO extent groups. **Table S5.** Ventilator setting and results of arterial blood gas measurement at the time of CT scans, before and after the 1st prone position. **Figure S1.** Axial computed tomographic (CT) image at the basal lung level of a 43-year old man with ARDS secondary to influenza pneumonia. The image was divided into sternal, central and vertebral sections. Each area was rated on a six-point scale of 0-5 based on the area of normal (N) lung tissue, consolidation (CO) or ground-glass opacification (GGO) present, with higher scores signifying a greater extent of lung involvement. **Figure S2.** Receiver operator characteristic (ROC) curve for various cutoff levels of total computed tomographic (CT) ground-glass opacification (GGO) scores predicting 60-day survival. **Figure S3.** Kaplan-Meier plots with log-rank test for the probability of 28-day survival, according to the response to prone positioning (Panel A), and total computed tomographic ground-glass-opacification (CT-GGO) scores (Panel B); Kaplan-Meier plots with log-rank test for the probability of ICU survival, according to the response to prone positioning (Panel C), and total computed tomographic ground-glass-opacification (CT-GGO) scores (Panel D).

## Data Availability

The datasets used and/or analyzed during the current study are available from the corresponding author on reasonable request.

## Abbreviations

ARDS: Acute respiratory distress syndrome
CI: Confidence interval
CT: Computed tomography
FiO_2_: Fraction of inspired oxygen
GGO: Ground glass opacification
ICU: Intensive care unit
IQR: Interquartile range
MV: Mechanical ventilation
PaO_2_: Arterial oxygen pressure
SAPS: Simplified Acute Physiology Score
SOFA: Sequential Organ Failure Assessment
P/F: Arterial oxygen pressure/fraction of inspired oxygen
