# Supplementary material for: Prognostic value of computed tomographic findings in acute respiratory distress syndrome and the response to prone positioning
Source: BMC Pulm Med. 2022 Feb 25;22:71. doi: 10.1186/s12890-022-01864-9 (PMC8874746; doi:10.1186/s12890-022-01864-9)
Supplement: Supplementary file 1 — Additional file 1. Table S1. Logistic regression to evaluate the association between prone-responders and dorsal-ventral differences in CT-consolidation scores at the lung base. Table S2. Ventilator setting and results of arterial blood gas measurement before and after the 1st prone position between responders and nonresponders. Table S3. Ventilator setting and results of arterial blood gas measurement before and after the 1st prone position between survivors and nonsurvivors. Table S4. Ventilator setting and results of arterial blood gas measurement before and after the 1st prone position between the high and low GGO extent groups. Table S5. Ventilator setting and results of arterial blood gas measurement at the time of CT scans, before and after the 1st prone position. Figure S1. Axial computed tomographic (CT) image at the basal lung level of a 43-year old man with ARDS secondary to influenza pneumonia. The image was divided into sternal, central and vertebral sections. Each area was rated on a six-point scale of 0-5 based on the area of normal (N) lung tissue, consolidation (CO) or ground-glass opacification (GGO) present, with higher scores signifying a greater extent of lung involvement. Figure S2. Receiver operator characteristic (ROC) curve for various cutoff levels of total computed tomographic (CT) ground-glass opacification (GGO) scores predicting 60-day survival. Figure S3. Kaplan-Meier plots with log-rank test for the probability of 28-day survival, according to the response to prone positioning (Panel A), and total computed tomographic ground-glass-opacification (CT-GGO) scores (Panel B); Kaplan-Meier plots with log-rank test for the probability of ICU survival, according to the response to prone positioning (Panel C), and total computed tomographic ground-glass-opacification (CT-GGO) scores (Panel D). [file 12890_2022_1864_MOESM1_ESM.docx]

**Table S1-** Logistic regression to evaluate the association between prone-responders and dorsal-ventral differences in CT-consolidation scores at the lung base

| Model | Dorsal-ventral difference in CT-consolidation scores | |
| --- | --- | --- |
|  | Odds ratio (95% CI) | p value |
| Model 1: unadjusted | 1.35 (1.12-1.61) | 0.001 |
| Model 2: adjusted for age and sex | 1.34 (1.11-1.60) | 0.002 |
| Model 3: further adjusted for BMI | 1.35 (1.12-1.63) | 0.002 |
| Model 4: further adjusted for the SAPS II score | 1.35 (1.12-1.63) | 0.002 |

Abbreviations: CT = computed tomography; SOFA = Sequential Organ Failure Assessment; SAPS = Simplified Acute Physiology Score.

**Table S2** Ventilator setting and results of arterial blood gas measurement before and after the 1^st^ prone position between responders and nonresponders

|  | Responders (n=68) | |  | | Nonresponders (n=28) | |  |
| --- | --- | --- | --- | --- | --- | --- | --- |
| Variable | Before 1^st^ Prone Positioning | After 1^st^ Prone Positioning | p value† | Before 1^st^ Prone Positioning | | After 1^st^ Prone Positioning | p value† |
| Tidal volume (ml) | 448.8±155.4 | 438.4±140.3 | 0.592 | 437.6±108.2 | | 393.8±98.0 | 0.086 |
| Tidal volume (ml/kg of PBW) | 7.9±2.7 | 7.7±2.3 | 0.573 | 8.2±7.3 | | 7.3±1.9 | 0.072 |
| Respiratory frequency (breaths/min) | 23.0±4.7 | 23.0±6.1 | 0.981 | 22.5±6.0 | | 22.1±6.9 | 0.783 |
| Dynamic driving pressure (cmH_2_O) | 15.3±2.7 | 15.7±3.3 | 0.452 | 15.7±4.1 | | 16.8±5.1 | 0.132 |
| PEEP (cmH_2_O) | 11.8±2.5 | 11.2±2.2 | 0.036 | 12.0±2.0 | | 11.6±2.3 | 0.185 |
| Plateau pressure (cmH_2_O) | 27.2±3.5 | 27.6±4.1 | 0.248 | 27.6±3.6 | | 28.2±4.0 | 0.230 |
| Mean airway pressure (cmH_2_O) | 17.7±2.9 | 17.6±3.4 | 0.839 | 17.9±2.5 | | 17.9±2.6 | 0.959 |
| Compliance (mL/cmH_2_O) | 30.6±12.6 | 28.6±13.8 | 0.213 | 30.6±12.7 | | 27.2±16.2 | 0.213 |
| Mechanical power (J/min) | 26.4±8.3 | 26.5±9.7 | 0.909 | 25.5±6.1 | | 23.6±7.7 | 0.214 |
| FiO_2_ (%) | 82.4±16.9 | 60.3±15.6 | <0.001 | 78.6±11.9 | | 75.5±13.9 | 0.222 |
| Arterial pH | 7.39±0.08 | 7.38±0.08 | 0.219 | 7.38±0.09 | | 7.35±0.08 | 0.227 |
| PaO_2_ (mmHg) | 73.8±17.2 | 105.6±32.6 | <0.001 | 78.8±16.8 | | 74.8±14.1 | 0.232 |
| PaCO_2_ (mmHg) | 39.2±11.7 | 42.6±16.8 | 0.103 | 46.1±21.6 | | 45.6±14.5 | 0.903 |
| PaO_2_:FiO_2_ | 95.1±34.8 | 184.4±67.5 | <0.001 | 103.5±31.4 | | 102.2±24.2 | 0.799 |
| Base Excess (mmol/L) | -1.6±5.8 | -1.4±4.9 | 0.822 | 0.7±5.8 | | -0.5±4.8 | 0.036 |

Data are presented as the no. (or %) or median (with the IQR).

† Paired t test before the 1^st^ prone positioning and after the 1^st^ prone positioning in responders

‡ Paired t test before the 1^st^ prone positioning and after the 1^st^ prone positioning in nonresponders

Abbreviations: PBW= predicted body weight; PEEP = positive end expiratory pressure

**Table S3** Ventilator setting and results of arterial blood gas measurement before and after the 1^st^ prone position between survivors and nonsurvivors

|  | Survivors (n=44) | |  | | Nonsurvivors (n=52) | |  |
| --- | --- | --- | --- | --- | --- | --- | --- |
| Variable | Before 1^st^ Prone Positioning | After 1^st^ Prone Positioning | p value† | Before 1^st^ Prone Positioning | | After 1^st^ Prone Positioning | p value† |
| Tidal volume (ml) | 421.9±103.6 | 455.6±142.9 | 0.106 | 465.5±167.4 | | 399.8±114.2 | 0.003 |
| Tidal volume (ml/ kg of PBW) | 7.4±1.9 | 7.9±2.4 | 0.098 | 8.4±2.9 | | 7.3±2.0 | 0.004 |
| Respiratory frequency (breaths/min) | 21.9±4.8 | 21.2±5.8 | 0.318 | 23.6±5.3 | | 24.1±6.4 | 0.641 |
| Dynamic driving pressure (cmH_2_O) | 15.1±3.2 | 15.2±3.5 | 0.911 | 15.6±3.2 | | 17.7±3.9 | <0.001 |
| PEEP (cmH_2_O) | 12.2±2.5 | 11.3±2.2 | 0.007 | 11.6±2.3 | | 11.3±2.2 | 0.444 |
| Plateau pressure (cmH_2_O) | 27.5±4.2 | 26.6±4.3 | 0.057 | 27.4±2.9 | | 29.4±3.5 | <0.001 |
| Mean airway pressure (cmH_2_O) | 17.8±2.9 | 16.6±2.9 | 0.010 | 17.8±2.7 | | 18.6±3.1 | 0.052 |
| Compliance (mL/cmH_2_O) | 29.6±11.6 | 32.6±27.9 | 0.089 | 31.5±13.5 | | 24.5±12.6 | <0.001 |
| Mechanical power (J/min) | 24.2±6.8 | 24.3±8.4 | 0.955 | 27.8±8. | | 26.9±9.7 | 0.494 |
| FiO_2_ (%) | 78.5±17.3 | 60.0±14.9 | <0.001 | 83.6±13.9 | | 68.8±17.0 | <0.001 |
| Arterial pH | 7.39±0.08 | 7.38±0.07 | 0.506 | 7.39±0.09 | | 7.36±0.08 | 0.106 |
| PaO_2_ (mmHg) | 74.8±18.3 | 98.9±31.7 | <0.001 | 75.7±16.2 | | 94.6±31.8 | <0.001 |
| PaCO_2_ (mmHg) | 38.6±9.9 | 39.7±8.1 | 0.413 | 43.4±18.7 | | 46.7±20.2 | 0.316 |
| PaO_2_:FiO_2_ | 102.2±40.7 | 175.4±70.2 | <0.001 | 93.6±26.7 | | 147.7±66.4 | <0.001 |
| Base Excess (mmol/L) | -2.1±6.2 | -2.0±4.2 | 0.926 | 0.1±5.5 | | -0.5±5.3 | 0.297 |

Data are presented as the no. (or %) or median (with the IQR).

† Paired t test before the 1^st^ prone positioning and after the 1^st^ prone positioning in survivors

‡ Paired t test before the 1^s^t prone positioning and after the 1^st^ prone positioning in nonsurvivors

Abbreviations: PBW= predicted body weight; PEEP = positive end expiratory pressure

**Table S4** Ventilator setting and results of arterial blood gas measurement before and after the 1^st^ prone position between the high and low GGO extent groups

|  | High GGO extent (n=53) | |  | | Low GGO extent (n=43) | |  |
| --- | --- | --- | --- | --- | --- | --- | --- |
| Variable | Before 1^st^ Prone Positioning | After 1^st^ Prone Positioning | p value† | Before 1^st^ Prone Positioning | | After 1^st^ Prone Positioning | p value† |
| Tidal volume (ml) | 454.5±163.4 | 418.9±129.3 | 0.132 | 434.5±113.2 | | 433.4±133.1 | 0.954 |
| Tidal volume (ml/kg of PBW) | 8.2±3.0 | 7.6±2.2 | 0.127 | 7.7±2.0 | | 7.6±2.1 | 0.814 |
| Respiratory frequency (breaths/min) | 23.9±5.1 | 24.8±6.6 | 0.291 | 21.6±4.8 | | 20.3±4.9 | 0.104 |
| Dynamic driving pressure (cmH_2_O) | 14.8±2.9 | 16.5±3.8 | 0.001 | 16.1±3.3 | | 16.6±4.1 | 0.259 |
| PEEP (cmH_2_O) | 12.0±2.3 | 11.2±2.0 | 0.011 | 11.7±2.5 | | 11.4±2.5 | 0.388 |
| Plateau pressure (cmH_2_O) | 26.9±3.1 | 27.6±3.5 | 0.080 | 27.7±4.0 | | 28.2±4.7 | 0.689 |
| Mean airway pressure (cmH_2_O) | 17.9±2.6 | 18.2±3.1 | 0.514 | 17.5±2.9 | | 17.0±3.2 | 0.315 |
| Compliance (mL/cmH_2_O) | 28.6±11.3 | 28.7±14.6 | 0.951 | 32.3±13.5 | | 27.8±14.5 | 0.031 |
| Mechanical power (J/min) | 27.3±8.3 | 27.6±9.5 | 0.828 | 24.8±6.7 | | 23.4±8.3 | 0.296 |
| FiO_2_ (%) | 81.3±16.2 | 66.3±17.3 | <0.001 | 81.2±15.3 | | 62.8±15.6 | <0.001 |
| Arterial pH | 7.38±0.09 | 7.36±0.08 | 0.316 | 7.40±0.08 | | 7.38±0.07 | 0.108 |
| PaO_2_ (mmHg) | 74.6±18.7 | 95.2±30.2 | <0.001 | 76.0±15.3 | | 98.4±33.6 | <0.001 |
| PaCO_2_ (mmHg) | 44.5±18.7 | 45.6±13.5 | 0.677 | 37.1±8.8 | | 40.9±2.9 | 0.162 |
| PaO_2_:FiO_2_ | 97.1±35.7 | 156.7±74.9 | <0.001 | 98.1±31.9 | | 164.9±62.1 | <0.001 |
| Base Excess (mmol/L) | 0.0±6.2 | -0.6±4.7 | 0.415 | -2.1±5.3 | | -1.9±5.0 | 0.795 |

Data are presented as No. (or %) or median (with the IQR)

† Paired t test before the 1^st^ prone positioning and after the 1^st^ prone position in the high GGO extent group

‡ Paired t test before the 1^st^ prone positioning and after the 1^st^ prone position in the low GGO extent group

Abbreviations: PBW= predicted body weight; PEEP = positive end expiratory pressure

**Table S5** Ventilator setting and results of arterial blood gas measurement at the time of CT scans, before and after the 1^st^ prone position

| Variable | Before CT | Before 1^st^ Prone | After 1^st^ Prone | p Value† | p Value‡ |
| --- | --- | --- | --- | --- | --- |
| Tidal volume (ml) | 438.9±120.5 | 439±131.2 | 425.4±130.5 | 0.998 | 0.195 |
| Tidal volume (ml/kg of PBW) | 7.9±2.3 | 7.9±2.5 | 7.6±2.2 | 0.945 | 0.153 |
| Respiratory frequency (breathes/min) | 23.2±6.2 | 23.0±5.2 | 22.8±6.3 | 0.792 | 0.860 |
| Dynamic driving pressure (cmH_2_O) | 15.5±2.8 | 15.6±3.3 | 16.6±3.9 | 0.664 | 0.001 |
| Plateau pressure (cmH_2_O) | 26.4±3.4 | 27.3±3.6 | 28.0±4.1 | 0.012 | 0.109 |
| PEEP (cmH_2_O) | 11.4±2.0 | 11.8±2.5 | 11.3±2.2 | 0.210 | 0.014 |
| Mean airway pressure (cmH_2_O) | 17.3±2.9 | 17.8±2.8 | 17.7±3.2 | 0.232 | 0.838 |
| Compliance (mL/cmH_2_O) | 29.5±10.4 | 29.8±11.9 | 28.0±14.5 | 0.839 | 0.071 |
| Mechanical power (J/min) | 25.5±7.8 | 26.2±7.7 | 25.7±9.2 | 0.565 | 0.619 |
| FiO_2_ (%) | 66.3±17.5 | 80.3±15.4 | 64.7±16.6 | <0.001 | <0.001 |
| Arterial pH | 7.40±0.08 | 7.39±0.08 | 7.37±0.08 | 0.259 | 0.085 |
| PaO_2_ (mmHg) | 92.1±45.0 | 76.6±15.8 | 96.6±31.6 | 0.003 | <0.001 |
| PaCO_2_ (mmHg) | 37.1±12.9 | 40.0±11.9 | 43.5±16.2 | 0.017 | 0.219 |
| PaO_2_:FiO_2_ | 147.1±71.6 | 97.6±33.9 | 160.4±69.2 | <0.001 | <0.001 |
| Base Excess (mmol/L) | -1.5±4.9 | -0.91±5.9 | -1.2±4.9 | 0.317 | 0.577 |

Data are presented as No. (or %) or median (with the IQR)

† Paired t test between the time of CT and before the 1^st^ prone positioning

‡ Paired t test before the 1^st^ prone position and after the 1^st^ prone position

Abbreviations: CT = computed tomography; PBW= predicted body weight; PEEP = positive end expiratory pressure

**Figure S1** Axial computed tomographic (CT) image at the basal lung level of a 43-year old man with ARDS secondary to influenza pneumonia. The image was divided into sternal, central and vertebral sections. Each area was rated on a six-point scale of 0-5 based on the area of normal (N) lung tissue, consolidation (CO) or ground-glass opacification (GGO) present, with higher scores signifying a greater extent of lung involvement.

**
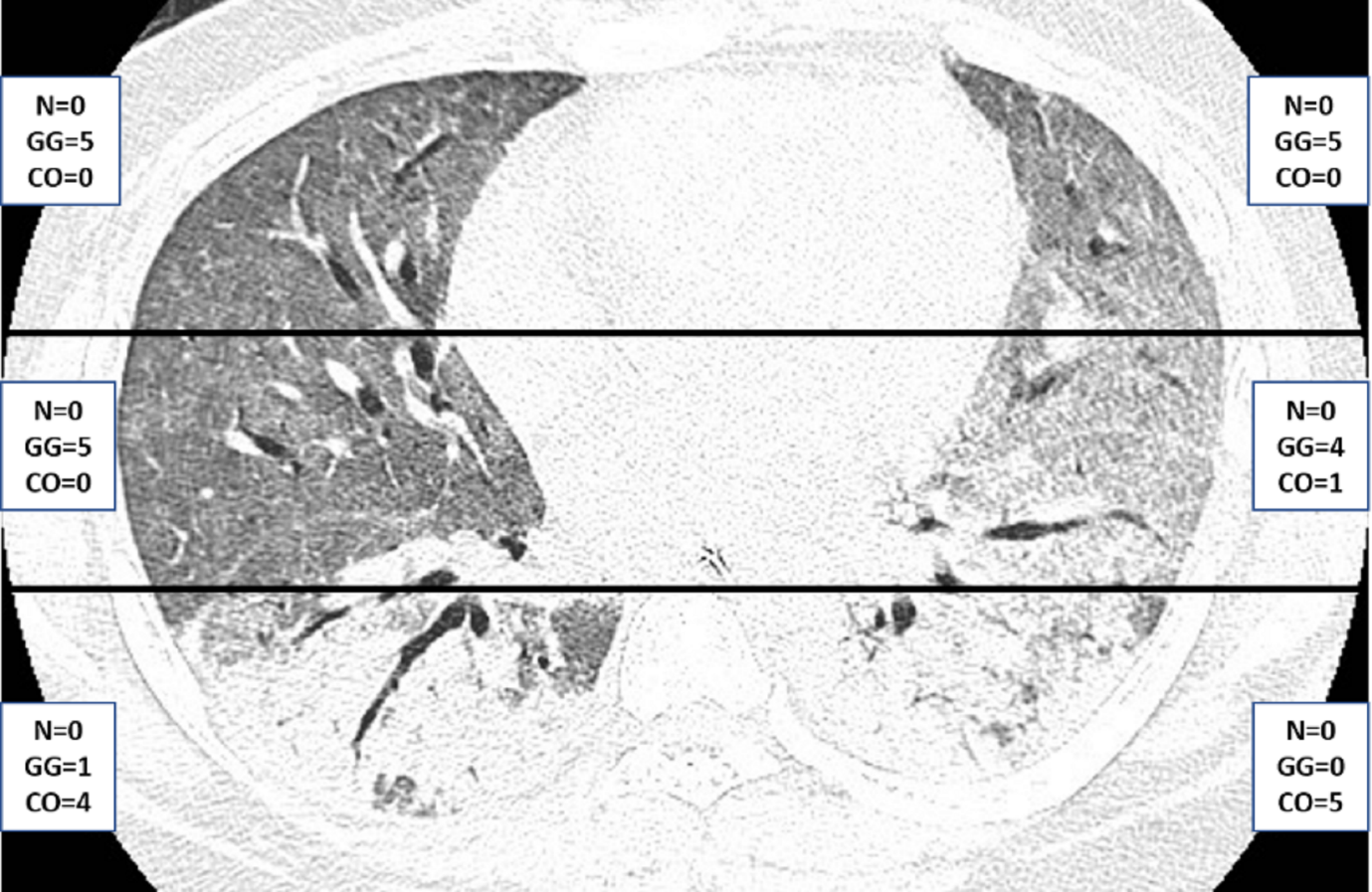
**

**Figure S2.** Receiver operator characteristic (ROC) curve for various cutoff levels of total computed tomographic (CT) ground-glass opacification (GGO) scores predicting 60-day survival.
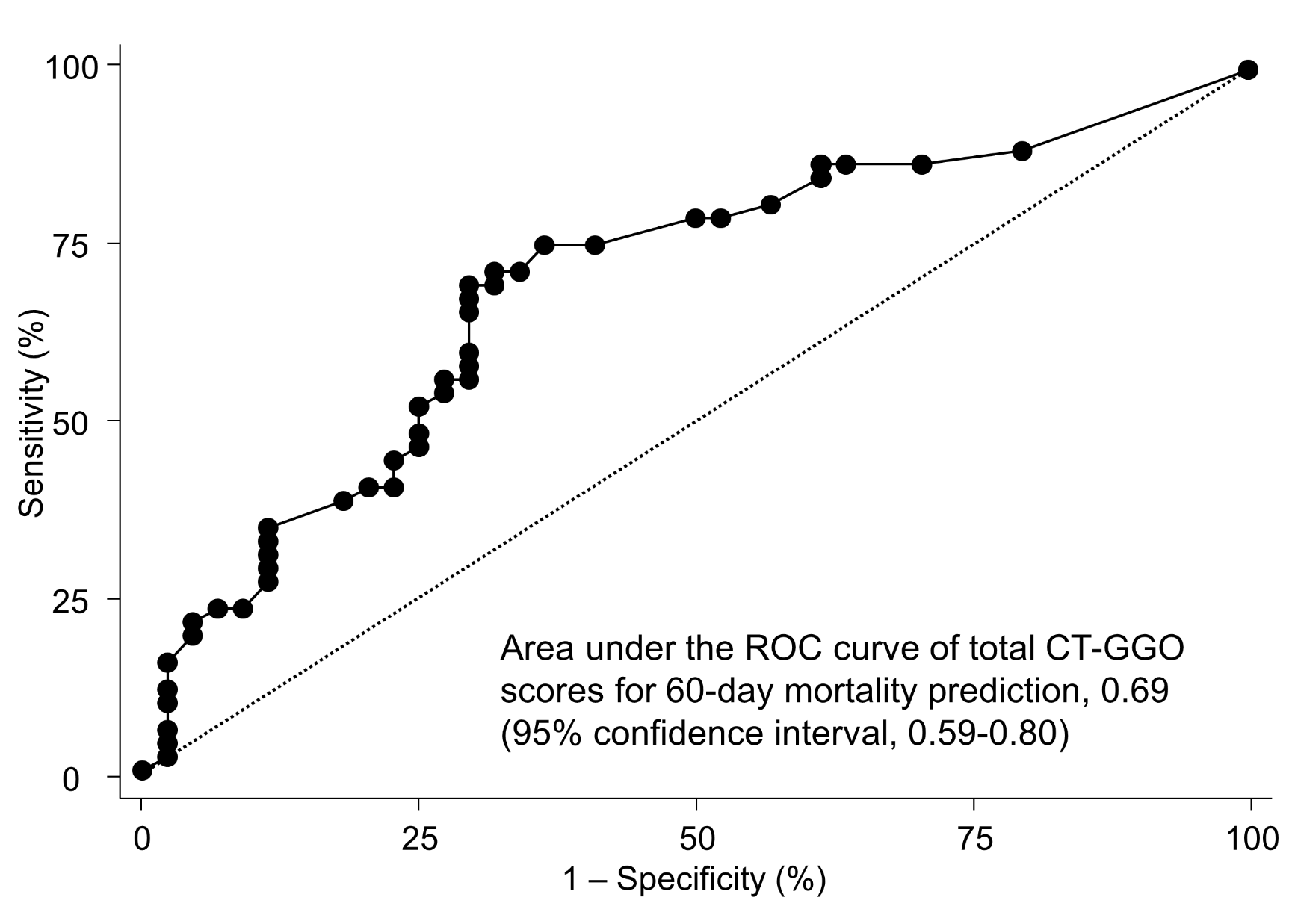


**Figure S3** Kaplan-Meier plots with log-rank test for the probability of 28-day survival, according to the response to prone positioning (Panel A), and total computed tomographic ground-glass-opacification (CT-GGO) scores (Panel B); Kaplan-Meier plots with log-rank test for the probability of ICU survival, according to the response to prone positioning (Panel C), and total computed tomographic ground-glass-opacification (CT-GGO) scores (Panel D)

**
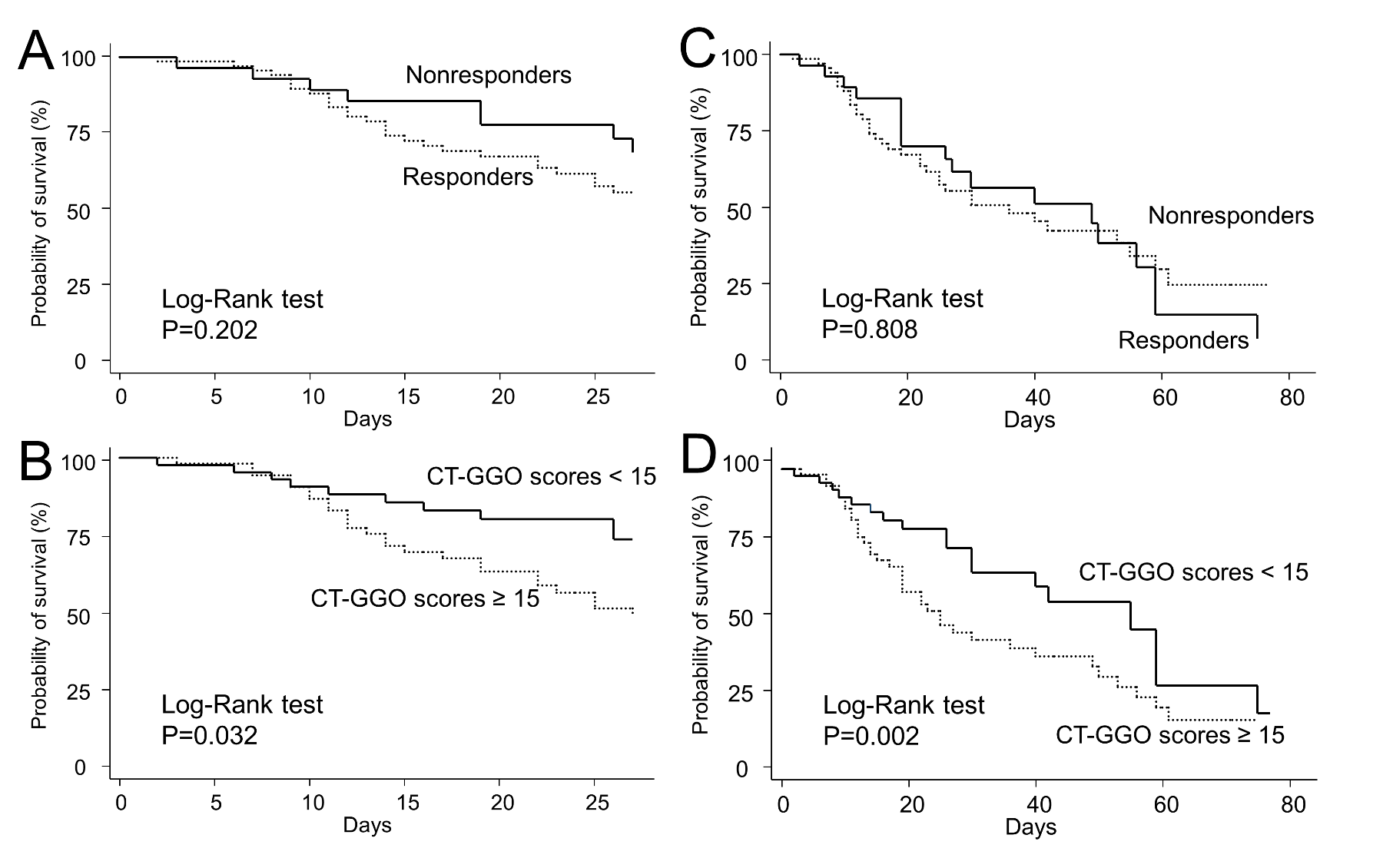
**
